# Adverse events and contributing factors in Chinese nursing homes: a multisite cross-sectional study

**DOI:** 10.3389/fpubh.2025.1518552

**Published:** 2025-02-25

**Authors:** Zhangan Wang, Xiang Qi, Ying Shi, Lu Shao, Wei Li, Xiyan Xie, Bei Wu, June Zhang

**Affiliations:** ^1^Department of Health Management, The People’s Hospital of Guangxi Zhuang Autonomous Region, Nanning, China; ^2^Rory Meyers College of Nursing, New York University, New York, NY, United States; ^3^School of Nursing, Sun Yat-sen University, Guangzhou, China; ^4^Department of Nursing, Home for the Aged Guangzhou, Guangzhou, China

**Keywords:** aging, long-term care, nursing home, risk management, safety management

## Abstract

**Background:**

Adverse events in nursing homes, which are unintended incidents causing unnecessary harm to older residents. Previous studies in Chinese populations often focused on adverse events in hospitals, rather than residents in nursing homes. Additionally, they tended to focus on single incident rather than multiple types of adverse events. This study aims to assess the occurrence and contributing factors of multiple adverse events perceived by staff in Chinese nursing homes.

**Methods:**

A cross-sectional survey was conducted among 691 frontline staff from 11 nursing homes in Southern China (August 2021–January 2022). Data were collected using a General Information Questionnaire, the Adverse Event Reporting Awareness Scale, and the Adverse Event Reporting Habit Scale. Logistic regression models were employed to analyze the contributing factors of adverse events.

**Results:**

A total of 13 types of adverse events were screened out in nursing homes, and 477 (69.0%) participants reported that the adverse events “had happened” in the past year. The most common events were falls, unplanned extubation, and pressure sores. Clinical staff were 2.06 times more likely than frontline workers to report adverse events (95% CI = 1.13–3.76). Increased awareness (OR = 1.24, 95% CI = 1.15–1.34) and habitual reporting of adverse events (OR = 1.04, 95% CI = 1.01–1.08) were positively associated with higher reporting rates.

**Conclusion:**

A significant proportion of staff reported adverse events, with clinical staff and those with better reporting habits noting higher occurrences. To enhance resident safety, nursing homes must prioritize preventing high-risk adverse events. Targeting frontline workers with lower reporting awareness and habits is crucial for effective interventions.

## 1 Introduction

The growing demand for long-term care services for older adults has become a daunting task for countries worldwide, especially in China, where population aging is particularly severe (1), coupled with the increasing prevalence of smaller family sizes, generational separation, and the weakening of traditional family care functions, more older adult individuals choose to reside in care institutions (2). As of 2023, 216.76 million older adults aged 65 and above lives in China, accounting for 15.4% of the total population (3). Aging is often accompanied by declining health, cognitive impairments, chronic diseases, and an increased mortality risk. These factors, along with smaller family sizes and the prevalence of intergenerational separation, make nursing homes a necessary option for many frail and disabled older individuals. As of the end of 2023, there are a total of 404,000 various types of senior care institutions and facilities in China, with a total of 8.23 million older adult care beds (3), representing a major industry in older adult care services. The Chinese government has made great efforts to improve the quality of care in nursing homes, such as establishing and enforcing national guidelines for operational standards and strengthening training for clinical staff and frontline workers (in Chinese nursing homes, clinical staff refers to professionals directly engaged in the medical care and treatment of residents, including physicians, nurses, rehabilitation therapists, psychologists, pharmacists, dietitians, and health managers. Frontline workers in nursing homes are personnel specialized in caring for older adults and providing daily services). However, challenges like staff shortages, inadequate training, and insufficient quality control persist, leading to a higher incidence of adverse events (AEs) (4).

AEs in nursing homes are unplanned, unexpected, or unwanted incidents occurring during older adult care that cause unintended and unnecessary harm to older residents, these include medication errors, falls, infections, and pressure sores, among others (4). AEs increase the probability of the admission of nursing home residents to the hospital, extend their hospital stay, increase the degree of suffering of older individuals and their families, and give rise to a public crisis of trust in older adult care service system (5). Recent studies highlight the prevalence and types of AEs in nursing homes, underscoring the critical need for effective risk management strategies. It is estimated that approximately 46.0% of nursing home residents and 11.0% of hospitalized patients in Finland are exposed to one or more AEs (6), with similar trends observed in China, albeit with limited data availability (7). A study conducted in New Zealand nursing homes indicated a high incidence rate of falls (13.0%) and pressure injuries (8.0%) (8). In the USA, approximately eight million AEs occurred annually in nursing homes and nearly a quarter of nursing home residents were admitted to hospitals because of iatrogenic infections. Literature review indicated that the incidence of AEs is high in nursing homes, but data on AEs in Chinese nursing home is scarce (7). Despite the recognized importance of error reporting in healthcare settings, the underreporting of AEs and near-miss events in nursing homes remains a substantial issue. Studies have shown that nursing home staff tend to report only severe AEs, leaving mild or moderate events largely unrecorded (9). This trend towards underreporting, were highlighted, and posed a significant challenge to effective error monitoring and prevention efforts. Inaccurate or incomplete data on AEs can lead to biased risk assessments and diminish staff’s ability to mitigate future incidents (10). While staff estimates are a common approach for data collection, offering a balance of quantity, speed, and cost-effectiveness, and they are subject to an acceptable level of bias (11).

Previous research has identified several factors that influence the occurrence and recognition of AEs, including facility characteristics, staff training, and AE reporting awareness and habits (12, 13). These factors are critical because accurate reporting and awareness are essential for understanding the true scope of AEs in nursing homes. A Korean study indicate that patient safety knowledge, safety climate, and just culture either directly or indirectly affected AEs reporting among military nurses (14). In addition, the staff’s educational background, competency, AEs reporting awareness, and reporting habits were also important contributing factors to the occurrence of AEs estimated by staff, AEs reporting awareness is closely related to reporting practice, and the top reporting barrier was “did not know what or how to report.” While existing research extensively covers AEs in nursing homes, a significant portion of this literature is based on western settings, which may not fully reflect the unique context of Chinese nursing homes. The cultural, organizational, and resource-related differences between western and Chinese long-term care facilities necessitate a context-specific investigation. Furthermore, many studies focus on single types of AEs, rather than a comprehensive assessment of multiple AEs and their interplay. Therefore, given the limited literature on AE reporting in Chinese nursing homes, insights from these studies are particularly valuable for understanding the dynamics in this context. Our study aims to fill these gaps by examining the occurrence and contributing factors of multiple AEs, utilizing staff perceptions within the Chinese nursing home environment. We specifically investigate the relationship between staff roles (clinical staff versus frontline workers), AE reporting awareness, AE reporting habits, and the organizational characteristics of nursing homes, which are novel aspects not thoroughly explored in previous research.

Previous research on AEs in long-term care facilities has often been limited to individual event types, with scant emphasis on quantifying multiple AEs as reported by nursing home staff (15). This study addresses two key gaps in the literature: (1) the lack of studies investigating multiple types of AEs concurrently in nursing homes, which limits comprehensive safety management strategies; and (2) the scarcity of research examining AEs and their contributing factors in non-western contexts, especially in Chinese nursing homes, where unique staffing, organizational, and cultural characteristics may influence AE occurrence and reporting. Previous research (16) identified key factors influencing AE occurrence and reporting in healthcare settings, with a focus on western and hospital contexts. This study extends those findings by examining how these factors manifest under the unique cultural and organizational environment of Chinese nursing homes. Specifically, we test the role of staff characteristics, reporting awareness, and habits in shaping AE trends across 13 types of incidents, providing both context-specific insights and broader implications for AE management in nursing homes globally. Studying multiple AEs in nursing homes is more important than studying individual events because it allows for a comprehensive understanding of the root causes of problems and the development of effective prevention and mitigation strategies. Comparatively, the body of research on AEs and contributing factors in long-term care facilities is considerably smaller than that for hospital settings, particularly outside of western contexts. Thus, our study seeks to address this gap by examining both the occurrence and contributing factors of various AEs in Chinese nursing homes. The primary research questions guiding this investigation are: (1) What are the types and frequencies of multiple AEs in Chinese nursing homes over the past year? (2) What are the factors influencing the occurrence of these AEs in this specific cultural and organizational context? Based on literature review (16), we hypothesized that (i) the characteristics of the staff may influence whether staff report AEs “occur or not”; (ii) staff’s AE reporting awareness and AE reporting habit may influence the reported occurrence of AEs.

## 2 Methods

### 2.1 Study design, setting and participants

This study was cross-sectional and followed the STROBE guidelines. All methods were carried out in accordance with relevant guidelines and regulations. The study was conducted from August 2021 to January 2022 in Guangzhou, the capital city of Guangdong province in China. In Guangzhou, 233 nursing homes are distributed in 11 districts, with 1,755,100 older adults, accounting for 18.4% of the city’s total population (17). To ensure geographical diversity, we recruited one nursing home from each district of the city and contacted the eligible staff from 11 selected nursing home using convenience sampling method.

The inclusion criteria for the participants were: (i) full-time front-line staff who could contact the residents directly in the nursing homes, including managers, physicians, nurses, frontline workers, rehabilitation therapists, and social workers; (ii) staff engaged in work in the nursing home for at least 1 year, as AEs of the previous year needed to be reported; (iii) staff participating in the survey voluntarily with informed consent. Exclusion criteria included individuals unable to complete the questionnaire independently due to literacy or technological barriers.

### 2.2 Data collection

**Figure 1:**
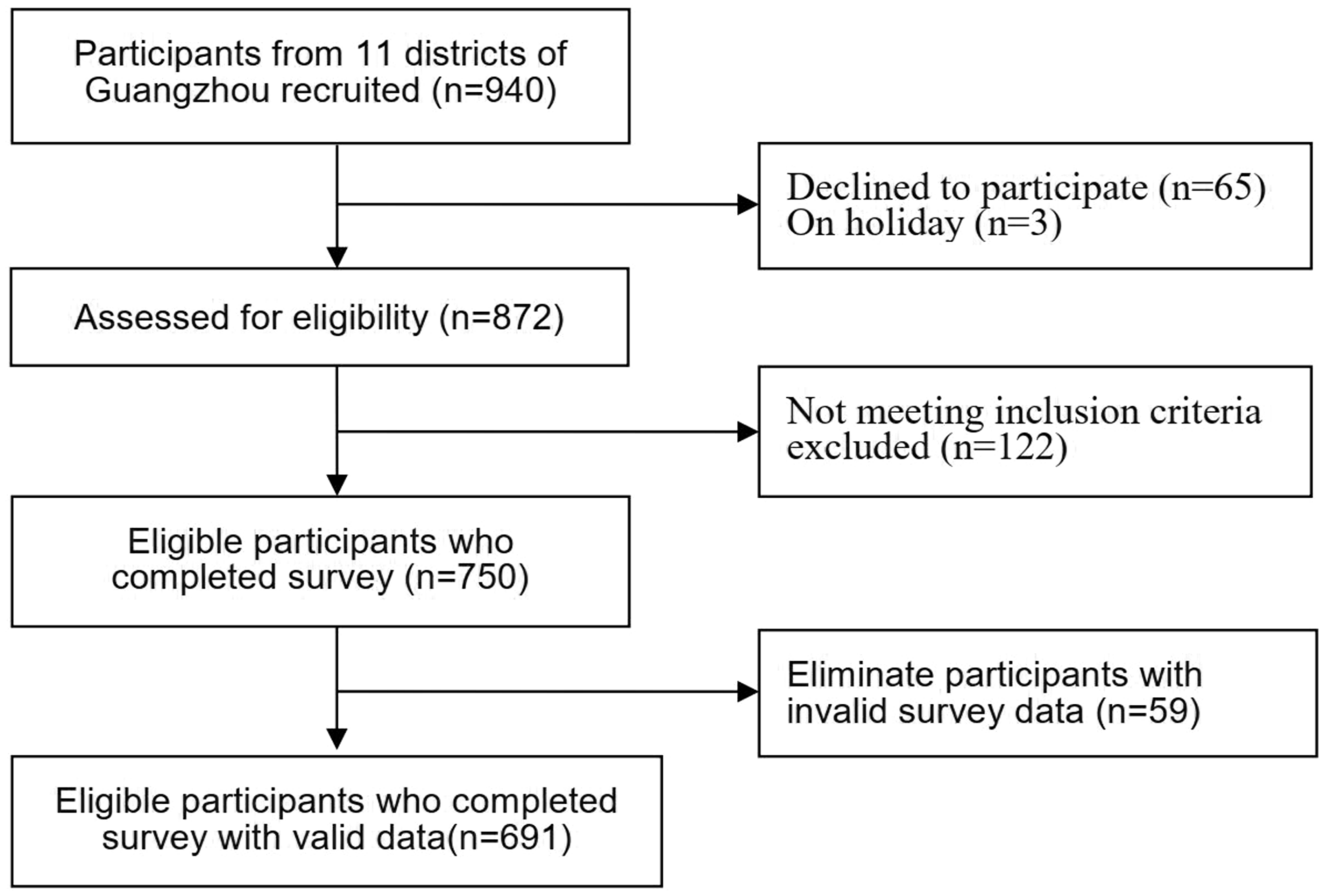
Flow diagram for the study.

Ethical approval for the study was obtained from the university’s ethics committee (Approval No. L2021XX1) and the administration of each participating nursing home. Recruitment of participants involved distributing introductory letters and placing informational posters in each facility to increase response rates. In light of COVID-19 precautions, data collection was conducted via an online questionnaire. A link provided access to a detailed study introduction, including objectives, research team information, estimated participation time (10–15 min), and assurances of data confidentiality and anonymity. Consent was obtained electronically; participants proceeded to the questionnaire only after affirmatively responding to a consent query. During the investigation, the phone number of a trained researcher at each participating facility was offered to provide explanation if necessary. Each participant could complete and submit the questionnaire once, using a mobile phone or computer, to prevent duplicate responses. To ensure validity and accuracy, two researchers independently recorded and checked all collected questionnaires. Of the 750 questionnaires received, 59 were discarded due to incompleteness or logical inconsistencies, resulting in a final tally of 691 valid questionnaires, yielding a response efficiency of 92.1%. The study’s flow diagram is presented in Figure 1.

### 2.3 Instruments

#### 2.3.1 The General Information Questionnaire

A self-designed general information questionnaire included three parts. The first part, completed by nursing home managers, focused on investigating the facility’s characteristics, including its scale, operation mode, the proportion of registered nurses among staff, staff to resident ratio, turnover rate (staff who left during the preceding 3 months divided by all staff), and nursing hours per resident day (the average hours worked by registered nurses and frontline workers, divided by the total number of residents per 24 h).

The second part focused on investigating the demographic and work-related information of the staff, including their gender, age, educational background, occupation, working experience, working hours per week, night shifts per week, income satisfaction, and job satisfaction from each participant. The selection of these personal characteristics was guided by prior research on AE occurrence and reporting in healthcare settings, as well as theoretical frameworks emphasizing the role of individual and occupational factors in safety culture. These variables were chosen to explore their potential impact on AE identification, reporting, and overall safety management in the unique context of Chinese nursing homes. Specifically, demographic factors such as age, gender, and educational background can influence the ability to identify AEs and attitudes toward reporting. Occupational factors like position, professional qualifications, years of experience, working hours per week, and night shifts can affect exposure to specific types of AEs and familiarity with reporting systems. Job satisfaction and income satisfaction are included as they are known to impact staff engagement and commitment, which can influence reporting rates.

The third part investigated AEs occurrence (had happened = 1, never happened = 0) and frequency in nursing homes. Prior to the investigation, one nursing home was selected for a pilot of all employees with more than 1 year’s experience (*n* = 51), comprising 3 physicians, 10 nurses, and 38 frontline workers. Analysis of pilot data confirmed that 13 types of AEs commonly occurred: fall, unplanned extubation, pressure sore, constraint complication, infection, choking or aspiration, self-injury or injury to others, medication error, burn, eating by mistake, getting lost, suicide, and other less common events (including older adult abuse, food poisoning, frostbite, etc.). Based on previous studies (11, 18), a 7-level rating scale was used to estimate the frequencies of these 13 types of AEs in the past year by staff in nursing homes, which were scored as “never happened = 0,” “several times a year = 1,” “once a month or less = 2,” “several times a month = 3,” “once a week = 4,” “several times a week = 5,” “every day = 6.”

#### 2.3.2 The Adverse Event Reporting Awareness Scale

The AE reporting awareness scale was initially used to evaluate the awareness of healthcare providers of AE reporting (19). The scale was translated into Chinese and revised by Tian (20). The scale consisted of eight items, and each item involves a statement of understanding about AE reporting. The participants were asked to make judgments according to the statements, such as “is there any AE reporting system in your institution,” and a response with “yes” was rated as “one point,” while “unknown” or “no” was rated as “zero.” The total score ranged from zero to eight points, and a higher score indicated a better awareness of AE reporting. The content validity index of the Chinese version of the scale was 0.96. In this study, the Cronbach’s *α* of the scale was 0.86.

#### 2.3.3 The Adverse Event Reporting Habit Scale

The scale to evaluate the AE reporting habit of the staff was developed by Vincent (21) and revised by Evans (19). The Chinese version of the scale was translated and revised by Tian (20) and is a 4-point rating scale consisting of five items (pressure sores, falls, medication errors, unplanned extubation and other AEs with detail). The Likert-4 was adopted as follows: 0 = not applicable, 1 = never, 2 = occasionally (reporting rate <50.0%), 3 = frequently (reporting rate ≥ 50.0%), 4 = always. The total score ranged from zero to 16 points, and a higher score indicated a better habit of AE reporting. The content validity index of the Chinese version of the scale was 1.00. In our study, the Cronbach’s *α* of the scale was 0.76.

### 2.4 Data analysis

Data analysis was conducted using SPSS (version 26). The participants’ demographic characteristics, work-related variables, and the frequency of AEs were described using descriptive statistic indicators such as frequency and percentage. Continuous variables roughly conforming to a normal distribution are presented by the mean and standard deviation (SD). A total of 20 variables were used in the analysis and categorized for this study as follows: institutional characteristics (6 variables: scale, operation mode，resident-staff ratio, proportion of registered nurses among staff, nursing hours per resident day provided by frontline workers and by registered nurses, respectively), participants’ characteristics (11 variables, including age, gender, education, post, professional qualifications, years of engaged in long-term care, night shifts per week, professional and technical personnel title, working hours per week, job satisfaction, income satisfaction), occurrence of AEs, AE reporting awareness, and AE reporting habits.

Following methodologies established in prior research (11, 18), the categories of frequencies of AEs were entered as response variables after the data were dichotomized into “never happened = 0” and “had happened = 1” (response of “several times a year,” “once a month or less,” “several times a month,” “once a week,” “several times a week,” or “every day”). Chi-square tests evaluated differences in the occurrence of staff-estimated AEs across various demographic and work-related characteristics, and *t*-tests were used to analyze the association between factors of interests and estimated AEs “had happened” or “never happened.” Univariate analyses were conducted to examine potential contributing factors of the occurrence of total AEs and each type of AE. To address potential confounding, we initially screened all 20 variables using univariate analysis and included variables that demonstrated a significant association (*p* < 0.05) with the outcome of interest in subsequent multivariate analyses. Subsequently, binary logistic regression models (Backward: Wald) were employed to analyze these factors’ association with the total occurrence of 13 types of AEs. In these models, the potential contributing factors were treated as independent variables, and the occurrence of each AE type fall, unplanned extubation, pressure sore, constraint complication, infection, choking or aspiration, self-injury or injury to others, medication error, burn, eating by mistake, getting lost, suicide, and other less common events (including older adult abuse, food poisoning, frostbite, etc.), as well as the total occurrence of 13 types of AEs, served as dependent variables. The threshold for statistical significance was set at *α* = 0.05. Furthermore, we also calculated the Pearson correlation matrix to evaluate the relationships between staff-estimated AE occurrence (dichotomized into “had happened” or “never happened”) and 20 influencing factors, and performed a one-way ANOVA to compare the average staff-estimated AE occurrence across several categorical factors, as described in the result section.

To address the limitation of not performing a formal power analysis before the study, we conducted a *post hoc* power analysis using observed effect sizes from our logistic regression models. With a sample size of 691 participants, a significance level of α = 0.05, and observed odds ratios for key variables (e.g., OR = 2.06 for clinical staff vs. frontline workers in reporting AEs), the study achieved a statistical power exceeding 80%, which is commonly considered sufficient to detect significant effects.

## 3 Results

### 3.1 General characteristics of participants

Table 1 presents the demographic and work-related characteristics of the 691 study participants. Approximately half (51.5%) were employed in privately operated nursing homes, with the remainder (48.5%) working in public social welfare facilities. The distribution of participants across facility sizes was as follows: 61.9% from large-scale, 29.1% from medium-scale, and 9.0% from small-scale nursing homes. The majority of participants were women (93.8%) and frontline workers (82.8%), with clinical staff (nurses, physicians, rehabilitation therapists, and social workers) constituting 17.2%. The age range was 19–60 years, with an average age of 46.2 ± 16.3 years. Notably, 98.4% of the participants worked over 40 h per week. The overall education level was low, with only 6.5% holding a bachelor’s degree or higher. The average nursing hours per resident per day were 2.4 ± 1.1 h, contributed by registered nurses (0.4 ± 0.4 h) and frontline workers (2.0 ± 0.7 h). The mean proportion of registered nurses among staff was 1:6.4, and the staff to resident ratio was 1:2.3. The annual staff turnover rate varied from 1.7 to 40.0%, averaging 11.4%.

**Table 1:** Occurrence differences of staff-estimated adverse events among different demographic and work-related characteristics (*N* = 691).

Characteristics	Frequency (*n*, %)	Never happened (*n*, %)	Had happened (*n*, %)	*χ* ^2^	*p*-value
Facility operation mode					
Public	335 (48.5)	81 (11.7)	254 (36.8)	12.386	<0.001^**^
Private	356 (51.5)	130 (18.8)	226 (32.7)		
Facility size					
Large (≥500 beds)	428 (61.9)	122 (17.7)	306 (44.3)	30.876	<0.001^**^
Medium (200–499 beds)	201 (29.1)	85 (12.3)	116 (16.8)		
Small (<200 beds)	62 (9.0)	4 (0.6)	58 (8.4)		
Gender					
Female	648 (93.8)	198 (28.7)	450 (65.1)	0.002	0.964
Male	43 (6.2)	13 (1.9)	30 (4.3)		
Age (years)					
<30	79 (11.4)	18 (2.6)	61 (8.8)	21.824	<0.001^**^
30–39	138 (20.0)	32 (4.6)	106 (15.3)		
40–49	363 (52.5)	109 (15.8)	254 (36.8)		
≥50	111 (16.1)	52 (7.5)	59 (8.5)		
Educational level					
Primary school	93 (13.5)	40 (5.8)	53 (7.7)	17.786	0.003^**^
Middle school	248 (35.9)	85 (12.3)	163 (23.6)		
High school/technical secondary school	194 (28.1)	53 (7.7)	141 (20.4)		
Junior college	111 (16.1)	27 (3.9)	84 (12.2)		
Bachelor’s degree or above	45 (6.5)	7 (1.0)	38 (5.5)		
Working experience (years)					
1–2	340 (49.2)	102 (14.8)	238 (34.4)	1.755	0.625
3–4	101 (14.6)	33 (4.8)	68 (9.8)		
5–9	99 (14.3)	26 (3.7)	73 (10.6)		
10~	151 (21.9)	50 (7.2)	101 (14.6)		
Occupation					
Older adult-care worker	572 (82.8)	189 (27.4)	383 (55.4)	9.838	0.002^**^
Healthcare professionals^b^	119 (17.2)	22 (3.2)	97 (14.0)		
Certified older adult-care worker (*n* = 572)					
Yes	448 (78.3)	142 (24.8)	306 (53.5)	1.691	0.193
No	124 (21.7)	47 (8.2)	77 (13.5)		
Working hours per week					
<40	11 (1.6)	4 (0.6)	7 (1.0)	9.427	0.051^a^
40–49	367 (53.1)	99 (14.3)	268 (38.8)		
≥50	313 (45.3)	108 (15.6)	205 (29.7)		
Night shifts per week					
≥2	454 (65.7)	130 (18.8)	324 (46.9)	2.255	0.133
≤1	237 (34.3)	81 (11.7)	156 (22.6)		
Work satisfaction					
Agree or strongly agree	257 (37.2)	87 (12.5)	172 (24.7)	2.920	0.232
Neutral	397 (57.5)	118 (17.0)	281 (40.4)		
Disagree or strongly disagree	37 (5.4)	8 (1.1)	30 (4.3)		
Income satisfaction					
Agree or strongly agree	184 (26.6)	67 (9.7)	117 (16.9)	4.963	0.084
Neutral	439 (63.5)	128 (18.5)	311 (45.0)		
Disagree or strongly disagree	68 (9.8)	16 (2.3)	52 (7.5)		

^*^p < 0.05 and ^**^p < 0.01.

a Fisher’s exact probability.

b Healthcare professionals: including nurses, physicians, rehabilitation therapists, and social workers.

### 3.2 The occurrence of adverse events estimated by staff

About 69.0% (*n* = 477) of participants estimated that AEs had occurred in the past year. The reported frequencies of these AEs ranged from “several times a year” (26.3%) to “once a week or less” (6.1%). No AE was reported as occurring “every day.” After combining 13 categories of frequencies of AEs into a binomial variable, the staff-estimated AEs that “had happened” in descending order as follows: falls (65.1%), unplanned extubation (33.6%), pressure sores (27.8%), choking or aspiration (18.4%), burns (16.4%), infection (15.2%), constraint complications (13.3%), medication errors (12.2%), self-injury or injury to others (11.7%), eating by mistake (5.5%), getting lost (4.3%), suicide (3.2%), and other events (older adult abuse, food poisoning, frostbite, etc., 7.2%), respectively (Table 2).

**Table 2:** Estimated adverse events in the past year among staff (*N* = 691).

Adverse events	Never happened (*n*, %)	Had happened (*n*, %)						
		Several times a year	Once a month or less	Several times a month	Once a week or less	Several times a week	Every day	Sum
Falls	241 (34.9)	250 (36.2)	109 (15.8)	63 (9.1)	15 (2.2)	13 (1.9)	0 (0.0)	450 (65.1)
Unplanned extubation	459 (66.4)	197 (28.5)	26 (3.8)	8 (1.2)	1 (0.1)	0 (0.0)	0 (0.0)	232 (33.6)
Pressure sores	499 (72.2)	182 (26.3)	9 (1.3)	1 (0.1)	0 (0.0)	0 (0.0)	0 (0.0)	192 (27.8)
Choking or aspiration	564 (81.6)	125 (18.1)	2 (0.3)	0 (0.0)	0 (0.0)	0 (0.0)	0 (0.0)	127 (18.4)
Burns	578 (83.6)	113 (16.4)	0 (0.0)	0 (0.0)	0 (0.0)	0 (0.0)	0 (0.0)	113 (16.4)
Infection	586 (84.8)	99 (14.3)	5 (0.7)	1 (0.1)	0 (0.0)	0 (0.0)	0 (0.0)	105 (15.2)
Constraint complications	599 (86.7)	83 (12.0)	7 (1.0)	2 (0.3)	0 (0.0)	0 (0.0)	0 (0.0)	92 (13.3)
Medication errors	607 (87.8)	82 (11.9)	2 (0.3)	0 (0.0)	0 (0.0)	0 (0.0)	0 (0.0)	84 (12.2)
Self-injury or injury to others	610 (88.3)	80 (11.6)	1 (0.1)	0 (0.0)	0 (0.0)	0 (0.0)	0 (0.0)	81 (11.7)
Eating by mistake	653 (94.5)	37 (5.4)	1 (0.1)	0 (0.0)	0 (0.0)	0 (0.0)	0 (0.0)	38 (5.5)
Getting lost	661 (95.7)	30 (4.3)	0 (0.0)	0 (0.0)	0 (0.0)	0 (0.0)	0 (0.0)	30 (4.3)
Suicide	669 (96.8)	22 (3.2)	0 (0.0)	0 (0.0)	0 (0.0)	0 (0.0)	0 (0.0)	22 (3.2)
Other events^a^	641 (92.8)	50 (7.2)	0 (0.0)	0 (0.0)	0 (0.0)	0 (0.0)	0 (0.0)	50 (7.2)
Sum	214 (31.0)	182 (26.3)	113 (16.4)	117 (16.9)	42 (6.1)	23 (3.3)	0 (0.0)	477 (69.0)

a Other events include older adult abuse, food poisoning, frostbite, etc.

### 3.3 Scores for the Adverse Event Reporting Awareness Scale and the Adverse Event Reporting Habit Scale and its association with occurrence of AEs

The mean scores for the AE Reporting Awareness Scale and the AE Reporting Habit Scale were (6.6 ± 2.3) and (9.5 ± 5.6), respectively. Furthermore, *t*-tests showed that the AE reporting awareness, AE reporting habits, the nursing hours per resident day provided by frontline workers, and the proportion of registered nurses among staff were potential factors influencing the occurrence of AEs estimated by participants (*p* < 0.05) (Table 3).

**Table 3:** Univariate analysis of continuous variables of the occurrence of 13 types of adverse events estimated by participants (*N* = 691).

	Adverse event reporting awareness		Adverse event reporting habit		Nursing hours per resident day provided by older adult care workers		Proportion of registered nurses among staff	
Occurrence	Never happened	Happened	Never happened	Happened	Never happened	Happened	Never happened	Happened
Mean	5.65	6.99	8.32	9.90	1.86	2.09	0.17	0.15
SD	2.66	1.96	6.15	5.42	0.70	0.73	0.08	0.06
*t*	−6.500		−2.640		−4.010		3.256	
*p*-value	<0.001^**^		0.009^**^		<0.001^**^		0.001^**^	

^*^p < 0.05 and ^**^p < 0.01.

### 3.4 Logistic regression analysis of the occurrence of adverse events estimated by participants

Prior to binary logistic regression, univariate analysis was performed to screen for potential factors affecting the occurrence of AEs. Chi-square tests revealed that the following variables were potential factors influencing the occurrence of AEs estimated by participants: the facility operation mode and the scale of the participating nursing home, the participants’ age, educational background, and occupation (Table 1). In the logistic regression model, whether participants were clinical staff or not, AE reporting awareness, and AE reporting habits were significant contributing factors of AEs estimated by participants. The logistic regression model correctly classified 69.2% of these observations (Table 4). Clinical staff were 2.06 times more likely than frontline workers to estimate that AEs had occurred (95% CI = 1.13–3.76, *p* = 0.019). As the mean scores for the AE reporting awareness increased by one point, the probability that participants estimated AEs as “had happened” increased by 23.8% (95% CI = 1.15–1.34, *p* < 0.001). Similarly, as the mean scores for the AE reporting habit increased by one point, the probability that participants estimated AEs as “had happened” increased by 4.2% (95% CI = 1.01–1.08, *p* = 0.017).

**Table 4:** Logistic regression analysis of the occurrence of adverse events estimated by participants (*N* = 691).

Variables	*β*	SE	Wald	OR	95% CI	*p*-value
Constant	−1.133	0.308	13.505	0.322		<0.001^**^
Occupation						
Older adult-care workers	0.000			1.000		
Healthcare professionals	0.722	0.308	5.488	2.058	(1.125–3.763)	0.019^*^
Adverse event reporting awareness	0.213	0.039	30.072	1.238	(1.147–1.335)	<0.001^**^
Adverse event reporting habit	0.042	0.017	5.712	1.042	(1.008–1.079)	0.017^*^

*β*, partial regression coefficient; SE, standard error of partial regression coefficient; OR, odds ratio; CI, confident interval. ^*^p < 0.05 and ^**^p < 0.01.

Binary logistic regression was also applied to each of the 13 AE types. For example, the occurrence of falls was significantly influenced by the scale of the nursing home, the occupation of the participant, and their AE reporting awareness. Compared to large-scale facilities, the likelihood of falls was lower in medium-scale (OR = 0.64, 95% CI = 0.44–0.93) and higher in small-scale homes (OR = 7.11, 95% CI = 2.50–20.22). Clinical staff were 2.64 times more likely than frontline workers to estimate falls (95% CI = 1.60–4.36, *p* < 0.001). Moreover, as the mean scores for the AE reporting awareness increased by one point, the probability that participants estimated falls “had happened” increased by 29.0% (95% CI = 1.19–1.39, *p* < 0.001).

## 4 Discussion

This study’s participant demographics, comprising nearly equal proportions from public (48.5%) and private (51.5%) nursing homes, align with national bed distribution trends in China, as reported by the Ministry of Civil Affairs (3). This similarity, coupled with congruent work-related characteristics such as a predominantly female workforce (93.8%), a low ratio of registered nurses to frontline caregivers, and an average of 2.4 nursing hours per resident per day, suggests that our sample broadly represents the target population of frontline staff in Chinese urban nursing homes. While these findings align with national data, our study also reveals nuances. For example, the registered nurse-to-staff ratio in our study (1:6.4) is slightly below the national standard of 1:5, suggesting possible staffing shortages, particularly in private facilities. Despite a high proportion of frontline workers, their limited training and qualifications, as indicated by our results, may hinder effective recognition and reporting of AEs. Addressing these issues is crucial for enhancing care quality and aligning with global benchmarks (6, 7). Similarly, the staff turnover rate of 11.4%, while matching the national average for urban areas, highlights a persistent challenge of workforce instability (7). These findings emphasize the need for policy interventions to address staffing levels and retention within the Chinese care sector. While direct international comparisons are limited by methodological differences, the general trends we observe in staffing ratios and turnover are consistent with global challenges in long-term care. Our research thus provides a critical, locally relevant perspective on the conditions impacting care delivery in China.

Our systematic approach to identifying 13 types of AEs in nursing homes, combining literature review and pilot study insights, the scale demonstrated strong reliability in assessing the frequency of these events. We discovered that staff-estimated AE occurrences (69.0%) far exceeded those reported administratively (about 5.0%), and it is much higher than the occurrences of AEs reported by hospital patients (23.6% of hospitalized patients experienced at least an AE in the United States) (22) (e.g., the prevalence of medical errors and AEs was 27.7 and 13.9%, respectively), among operating room nurses in China (23), suggesting AEs underreporting, particularly of non-serious AEs. The predominance of falls among reported AEs resonates with previous findings (7, 15). Surprisingly, a significant proportion of staff estimated unplanned extubation occurrences, which may reflect the high number of disabled older residents in Chinese nursing homes. Pressure sores, a well-documented issue in such settings, ranked third in frequency. Our results were consistent with the Chinese policy that the national standard for older adult care services, implemented in 2022 by the Chinese government (24), highlights the ongoing need to enhance safety in aged care.

A notable finding is that 31.0% of staff reported no AEs, possibly reflecting limited AE recognition skills and a cultural tendency to report AEs passively (“*Bao xi bu bao you*” in Chinese) (25). Under the traditional Chinese Confucianism culture context, Chinese staff are usually modest, courteous, and pay more attention to interpersonal relations, and they prefer to avoid trouble whenever possible (“Duo Yi Shi Bu Ru Shao Yi Shi” in Chinese), even if the staff know that their participation of the study would be kept confidential. In a previous study, it was also shown that a “punitive atmosphere” and “poor reporting management” are the most critical obstacles to reporting AEs in nursing homes in China (10). Other previous studies indicated that perceptions of fear of retribution and blaming, lack of feedback, and comfort level of challenging someone authoritative produced the greatest obstacles to AE reporting (26). In addition, systemic issues, such as inadequate reporting systems and insufficient training, contribute to underreporting. These findings suggest that improving AE reporting in nursing homes requires addressing both individual and organizational barriers (27). Another explanation for the staff in our study not reporting AEs may be the insensitivity of AEs to older adults, for example, some staff or frontline workers do not consider falls that do not fall to the ground as AE because of their limited understanding and judgment of AEs. Our findings underscore the need for nursing home managers to foster a non-punitive safety culture and enhance staff competence and sensitivity in AE identification.

Consistent with our hypothesis, clinical staff were more likely (OR = 2.06) to report AEs than frontline workers. This finding aligns with a Finnish study (4) and may be attributed to clinical staff’ higher educational and training levels, enabling better AE detection (14). Furthermore, the lower frequency of AE reporting among frontline workers compared to clinical staff may stem from several factors. These include limited formal education and specific training on AEs, less awareness of AE definitions and reporting systems, and cultural barriers, such as fear of blame or retribution. Additionally, frontline workers may lack the confidence to report AEs, particularly non-critical incidents, due to an insufficient understanding of what constitutes an AE. These factors highlight a crucial need for targeted interventions.

Therefore, increasing the ratio of clinical staff to frontline workers and improving training on AE recognition can enhance safety and care quality. Additionally, we found that AE reporting awareness and habits emerged as critical predictors of AE occurrence estimation. Staff with higher reporting awareness and habits estimated more AEs, indicating the importance of skills, knowledge, and training in effective incident reporting (23). A study revealed that all reports affecting resident safety and quality of care decline in nursing homes were related to systemic factors (28), therefore, from an organizational perspective, nursing home managers should create a positive, non-punitive safety environment to encourage AEs reporting. Furthermore, at the individual level, the staff should be trained to improve their AE reporting awareness and establish good AE reporting habits to optimize care of older residents in nursing homes.

This study identified contributing factors that may affect the reporting of falls. As shown in Table 4, the reporting of fall events depends on factors such as the size of the nursing home, the occupation of employees, and the awareness of AEs reporting. Specifically, compared to large institutions (≥500 beds), medium-sized institutions (200–499 beds) have significantly lower fall reporting frequency, which may be due to relatively good environmental facilities and personnel allocation in the large institutions. The reported fall incidents in small institutions (<200 beds) were significantly higher than those in large long-term care facilities, which may be related to the flat and humanized management model in small institutions. Professionals report more falls than older adult caregivers because they have received professional training and have a better understanding and knowledge towards falls and other AEs (see Table 5).

**Table 5:** Logistic regression analysis of the occurrence of falls estimated by participants (*N* = 691).

Variables	*β*	SE	Wald	OR	95% CI	*p*-value
Constant	−1.131	0.292	14.990	0.323		<0.001^**^
Facility scale						
Large	0.000		21.315	1.000		<0.001^**^
Medium	−0.447	0.190	5.532	0.639	(0.440–0.928)	0.019^*^
Small	1.962	0.533	13.528	7.110	(2.500–20.222)	<0.001^**^
Occupation						
Older adult-care workers	0.000			1.000		
Healthcare professionals	0.972	0.255	14.544	2.643	(1.604–4.356)	<0.001^**^
Adverse event reporting awareness	0.253	0.039	41.575	1.288	(1.192–1.390)	<0.001^**^

*β*, partial regression coefficient; SE, standard error of partial regression coefficient; OR, odds ratio; CI, class interval. ^*^p < 0.05 and ^**^p < 0.01.

This study underscores the critical need for development of policies and clinical guidelines to enhance nursing home safety. Politically, our findings call for policymakers to prioritize nursing home safety by developing targeted policies, enforcing robust regulatory frameworks that include mandatory adverse event reporting systems, and increasing government investment in staff training and education. Such measures are crucial for ensuring consistent safety practices and improving overall outcomes (29). Practically, this research highlights the importance of comprehensive training programs focused on adverse event recognition and reporting, using simplified reporting platforms, and fostering a non-punitive safety culture. Furthermore, nursing homes must invest in infrastructure and resources, such as adequate staffing and updated medical equipment, to prevent common AEs like falls and pressure sores. Addressing these practical aspects is essential for creating a safer and more effective care environment.

To address the lower propensity of frontline workers to report AEs, several practical strategies should be implemented. Firstly, targeted training programs should be designed and implemented to address adverse event identification and reporting processes, specifically tailored to meet the unique needs and varying educational levels of frontline workers. Nursing home managers should prioritize the development of team-focused education and training initiatives, specifically adapted for nursing staff with longer tenure and lower levels of formal education, to enhance their engagement and competency in safety practices (30). This should include the use of real-life case studies and practical exercises to enhance comprehension and applicability (31). Secondly, fostering a non-punitive reporting culture is essential, which can be achieved by creating anonymous reporting systems, recognizing and rewarding staff contributions to safety, and providing supportive feedback to encourage open communication. Thirdly, implementing feedback mechanisms is crucial, where staff receive timely information on how their AE reports are used to improve resident safety, reinforcing their role in safety management (32). Fourthly, strengthening leadership support is also vital, with nursing home managers actively promoting AE reporting and ensuring policies align with a supportive environment. Simultaneously, the establishment of a streamlined, non-punitive reporting system is essential to foster a positive safety management climate and encourage transparent communication regarding AEs (30). Finally, developing clear, simplified, and accessible AE reporting guidelines and tools, such as checklists or digital apps, will reduce the complexity and time required for reporting. These strategies aim to address the key barriers such as lack of awareness, fear of retribution, and limited understanding of AE definitions, and will help improve the reporting behaviors of staff with a lower propensity to report, ultimately enhancing safety and care quality in nursing homes.

This study has several limitations. Firstly, the cross-sectional nature of this study only allows us to examine the relationship between staff characteristics and the occurrence of AEs. Future longitudinal or experimental studies are needed to test causal pathways and evaluate interventions aimed at improving AE reporting and prevention. Secondly, the study outcome was measured based on staff-reported information. These results relied on self-reported data, which may be subject to recall bias. Staff might have under- or over-estimated the frequency of certain events due to memory limitations or social desirability bias. Future studies should consider integrating objective data sources, such as incident reports and observational audits, to validate self-reported findings and reduce recall bias. Thirdly, excluding participants due to their inability to operate a smartphone or limited education who unable to understand the electronic questionnaires independently may have introduced sampling bias. Fourthly, the results of this study, while providing valuable insights into the occurrence and contributing factors of AEs in nursing homes, may not fully generalize to less developed regions as we only recruited participants from Guangzhou, a relatively developed city in China. Future research should recruit samples from diverse socio-economic and geographical contexts to ensure a more comprehensive understanding. Furthermore, while we have taken steps to control for measured confounders using univariate screening and subsequent multivariate regression, we acknowledge that residual confounding cannot be entirely ruled out. This is particularly true for unmeasured variables, such as organizational culture and team dynamics within each facility, which might influence adverse event reporting practices.

In addition, future research should incorporate qualitative methods, such as interviews or focus group discussions, to explore the cultural, organizational, and personal barriers contributing to AE underreporting. This would provide a more holistic understanding and inform targeted interventions.

### 4.1 Practical applications

The insights from this research have significant implications for enhancing safety management and care quality in nursing homes. It is vital for these facilities to prioritize the prevention of AEs, especially those frequently occurring and posing substantial risks to the safety and health of older adult residents, such as falls, unplanned extubation, and pressure sores. Implementing strategies to prevent falls should be a primary focus in the routine operations of nursing homes. Furthermore, targeted interventions are needed for frontline workers who exhibit lower levels of AE reporting awareness and habits, as they represent a crucial group for improving AE management and prevention.

## 5 Conclusion

This study found that a majority of staff (69%) in Chinese nursing homes estimated that AEs had occurred within the past year, with falls, unplanned extubation, and pressure sores being the most commonly reported events. Clinical staff and staff with higher AE reporting awareness and better reporting habits tended to report a greater number of AEs. These findings align with existing literature indicating the high prevalence of falls and pressure sores in long-term care settings globally. However, the study also reveals unique challenges specific to Chinese nursing homes. The high incidence of unplanned extubation may reflect the large number of disabled older residents, a challenge that demands targeted strategies. Therefore, enhancing the AE reporting awareness and habits among frontline workers, particularly those currently exhibiting lower levels in these areas, is essential. Cultural factors, such as a tendency toward reporting only the good news and not the bad (“*Bao Xi Bu Bao You*”), may contribute to the underreporting among frontline workers (25). Addressing these cultural influences through training and creating a supportive reporting environment is crucial. Focusing on this group for further training and intervention could significantly contribute to improving the overall safety and quality of care in nursing homes.

## Data Availability

The raw data supporting the conclusions of this article will be made available by the authors, without undue reservation.
